# Immature Insect Assemblages from the Early Cretaceous (Purbeck/Wealden) of Southern England

**DOI:** 10.3390/insects12100942

**Published:** 2021-10-17

**Authors:** Robert A. Coram, Edmund A. Jarzembowski

**Affiliations:** 1 School of Earth Sciences, University of Bristol, Bristol BS8 1RJ, UK; 2 State Key Laboratory of Palaeobiology and Stratigraphy, Nanjing Institute of Geology and Palaeontology and Center for Excellence in Life and Paleoenvironment, Chinese Academy of Sciences, Nanjing 210008, China; 3 Department of Earth Sciences, The Natural History Museum, Cromwell Road, London SW7 5BD, UK

**Keywords:** Early Cretaceous, Purbeck, Wealden, palaeosalinity, fluvio-lagoonal, aquatic insects, nymphs, larvae, trace fossils, taphonomy

## Abstract

**Simple Summary:**

The non-marine Lower Cretaceous Purbeck and Wealden rocks of southern England provide an important record of insects that lived alongside the dinosaurs. Most fossil remains are those of adult insects from orders alive today, but immature insects and their trace fossils occur in the same deposits. Terrestrial immatures comprise mostly sessile nymphs of true bugs, whereas the aquatic immature fauna is represented by stoneflies and mayflies (rarely), dragonflies (uncommonly), true bugs and true flies (often common in the Purbeck), and the cases of caddisflies (locally common in the Wealden). These fossils help to shed light on the local palaeoenvironment, such as the salinity of water bodies, as well as on the processes that lead to the fossilization of generally fragile insect remains.

**Abstract:**

The record of immature insects from the non-marine Purbeck and Wealden groups (Lower Cretaceous) of southern England is reviewed and expanded. Fossils of adult terrestrial insects are locally common, but terrestrial immature remains are restricted to transported hemipterans, most of which are sessile nymphs or puparia resembling those of extant whiteflies (Aleyrodidae). Remains of immature aquatic insects are more diverse and comprise the extant orders Plecoptera, Ephemeroptera, Odonata, Trichoptera, Hemiptera and Diptera. The Trichoptera are represented by larval cases constructed from a variety of materials corresponding to several ichnogenera. The Wealden immature insects were preserved in predominantly freshwater fluvial settings, whereas the Purbeck ones occur in lagoonal palaeoenvironments, ranging in salinity from brackish to hypersaline. The composition of aquatic immature insect faunas in the latter offers potential for palaeosalinity analysis, although there are complicating factors relating to habitat stability. Uncommon trace fossils such as beetle borings in wood provide evidence of immature insects not represented by body fossils.

## 1. Introduction and Geological Setting

The Early Cretaceous, somewhat past the mid-point of the Mesozoic Era (‘age of dinosaurs’) and succeeding the Jurassic Period, was a time when the insect fauna was composed virtually entirely of extant orders but still contained archaic elements. It also lacked groups such as butterflies and bees now mostly associated with flowering plants, since angiosperms were yet to dominate terrestrial ecosystems.

**Figure 1:**
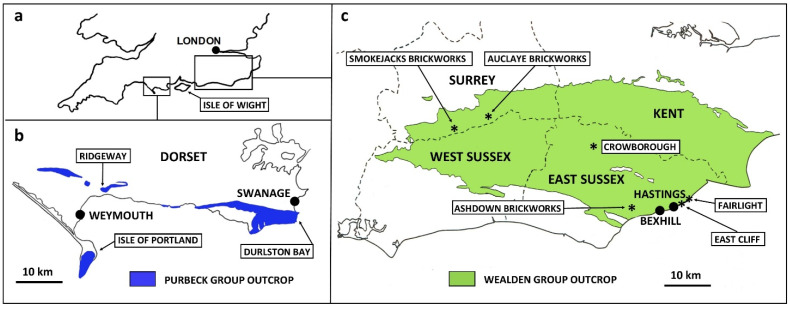
Locations of sites mentioned in text yielding Early Cretaceous immature insect fossils. (**a**) Outline map of southern England; (**b**) Purbeck Group outcrop of Dorset; (**c**) Wealden Group outcrop of southeast England.

A prolonged regression at the close of the Jurassic (end-Tithonian) led, in southern England, to the normal marine conditions that had prevailed for most of that period giving way to predominantly lagoonal and then fluvial depositional environments that persisted until marine conditions returned in the Cretaceous, around the early Aptian, after approximately 22 million years [1]. The corresponding deposits, the Purbeck and Wealden groups, respectively [2] (Figure 1), were laid down in variably inundated lowland settings that lay among a complex of isolated massifs bordered by the widening Protoatlantic to the west, the Boreal Sea to the northeast and the Tethys Ocean to the south. Both groups yield fossil remains of mostly non-marine organisms. The aquatic biota includes ostracods, molluscs and fish, and the terrestrial plants, reptiles and more rarely mammals. Insect remains can also be abundant at certain levels in both groups. Over 300 Purbeck species have been named, roughly double the number from the Wealden, which has a shorter study history; many collected specimens, however, remain to be described (especially the hyperdiverse beetles), and more insect species no doubt await discovery [3,4]. Among these finds are immature insect remains, which are generally uncommon. Here, we review the existing literature concerning these fossils and augment it with new material and discussion.

**Figure 2:**
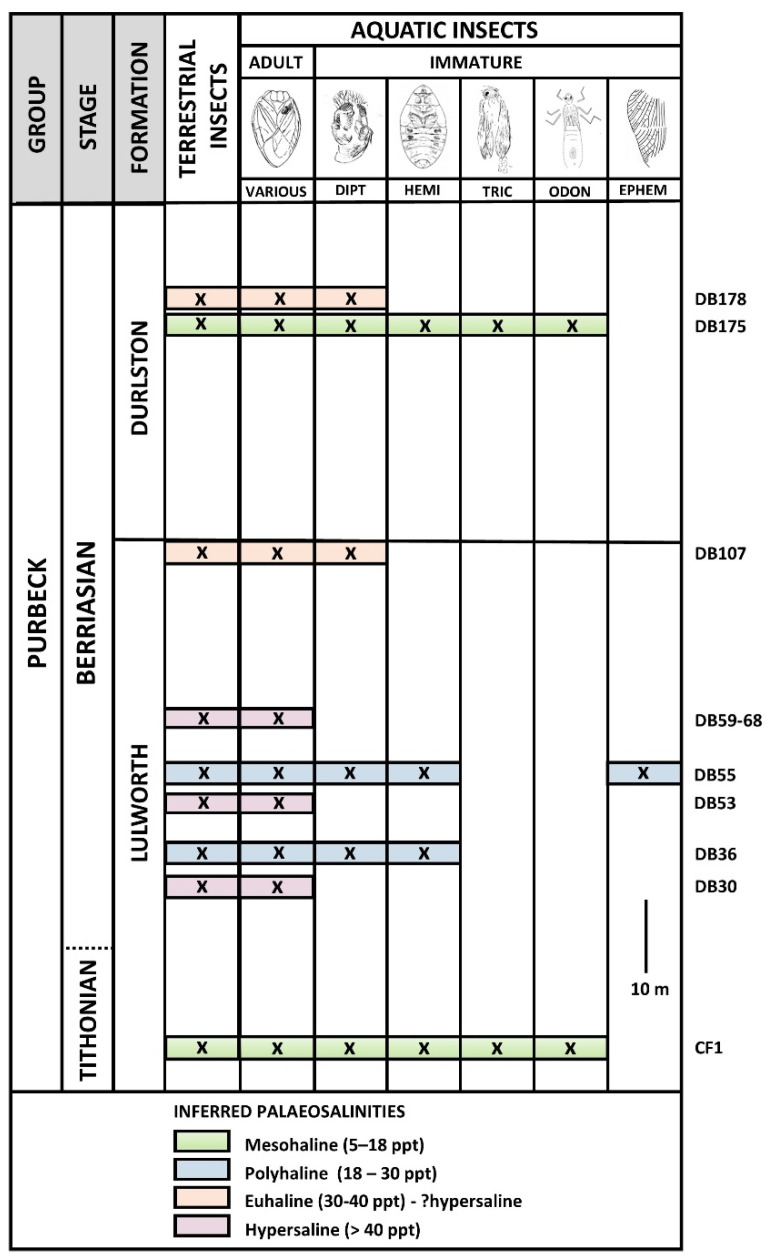
Distribution of immature insect fossils in the Purbeck Group of Durlston Bay (most productive horizons only shown). Inferred Purbeck palaeosalinities are based on the preserved immature aquatic insect fauna. Bed numbering from Clements [9] and West [10]. Terrestrial insects include whitefly-like nymphal Hemiptera, found in most horizons.

The Purbeck Group is a regressive rift sequence lying between the marine Jurassic Portland Group and the overlying Cretaceous Wealden Group. It essentially corresponds to the Berriasian Stage of the Lower Cretaceous, although the Jurassic/Cretaceous boundary (dated at 143 ma [1]) probably lies within the lower part of the succession (e.g., [5,6,7]), and the highest Purbeck may be earliest Valanginian [5]. The group is divided into the lower Lulworth and upper Durlston formations [2] (Figure 2).

The Purbeck sediments comprise predominantly lacustrine and lagoonal limestones and mudrocks deposited in a seasonally semi-arid to arid climate [8]. Purbeck strata are well exposed in numerous coastal and quarry sections in east Dorset (Figure 1), and these are the primary source of insect fossils. The two most significance sites for immature insects (comprising both terrestrial and aquatic taxa) are Durlston Bay and the Isle of Portland. The coastal outcrop at Durlston Bay, near Swanage (National Grid Reference SZ 040786—SZ 036772) is the type section of the group and, at *c*. 120 m, the thickest onshore section. The Isle of Portland (e.g., at NGR SY 691702) shows only the lower part of the Purbeck succession, in cliff sections and as quarry overburden. 

The succeeding Wealden sediments, mostly fluvial in origin and comprising mainly mudstones and sandstones, were deposited under a more humid climate than the Purbeck and, although prone to evaporation, lack the extreme aridity and hypersalinity experienced earlier [8]. 

**Figure 3:**
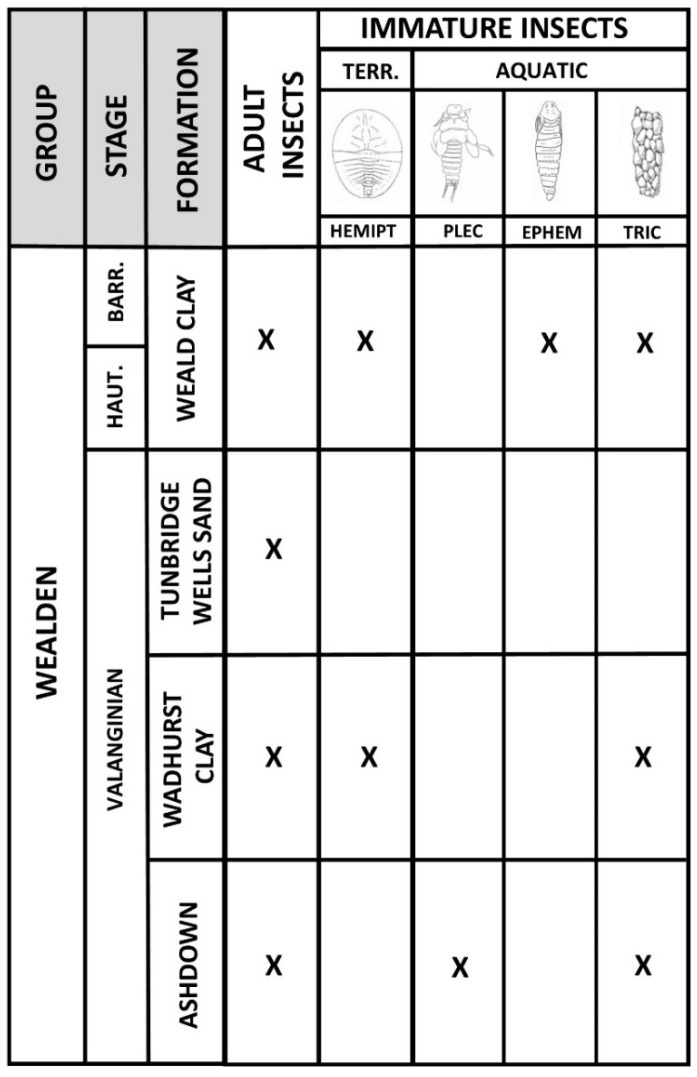
Distribution of insect fossils in the Wealden Group of southeast England. Formation thicknesses not to scale due to being highly variable at outcrop [2].

Wealden strata are exposed in coastal and quarry sections in southern and southeast England [11] (Figure 1), the latter being best known for insect fossils. In southeast England, the group comprises, from bottom to top, the Ashdown, Wadhurst Clay, Tunbridge Wells Sand and Weald Clay formations [2], ranging from the Valanginian to approximately basal Aptian stages of the Cretaceous (138–121 mya [1]; Figure 3). In the Isle of Wight, it comprises the Wessex and Vectis formations (Barremian to basal Aptian at outcrop).

A number of sites in southeast England have yielded fossils of immature insects. One of the most significant of these is East Cliff in the classical lower Wealden (Ashdown Formation) section at Hastings in East Sussex (NGR TQ 830096). Another is the Ashdown brickworks (NGR TQ 720095), Bexhill, also in the lower Wealden of East Sussex, additionally exposing the slightly younger Wadhurst Clay Formation, reported herein. Both of these principal sites (and others, including the Isle of Wight) have yielded immature aquatic insects, but occasional terrestrial immatures also occur at Bexhill and in the upper Wealden in south Surrey at the Smokejacks (NGR TQ 115372) and former Auclaye brickworks (NGR TQ 170388). The lower Wealden near Hastings at Fairlight has previously yielded a unique Early Cretaceous immature aquatic insect (stonefly, see below) [12]. With the exception of East Cliff, where the insects occur in arenaceous sediments, the insects are found in concretions in the argillaceous units: phosphatic at Auclaye and sideritic at Fairlight and Smokejacks; both principal lithologies are available due to faulting at Bexhill.

## 2. Materials and Methods

Figured material was collected by R.A.C. (Purbeck) and E.A.J. (Wealden), except where indicated in the figure captions. Repositories for deposited material are as follows: Bexhill Museum, East Sussex, UK (BEXHM); Maidstone Museum and Bentlif Art Gallery, Maidstone, Kent, UK (MNEMG); Natural History Museum, London, UK (NHMUK); University of Bristol, Bristol, UK (BRSUG). Some of the specimens (indicated in the figure captions) are much clearer images of previously published monochrome photographs. The remaining fossils are figured and discussed here for the first time.

Photographs of Purbeck insects were taken with an Olympus E-420 camera attached to a Zeiss Stemi SV6 stereomicroscope; Wealden insects were illustrated either from archive pictures in the collection of E.A.J. or photographed with an Olympus TG-4 digital camera through a Meiji Techno EMZ-5TR Trinocular stereomicroscope illuminated by FL150 fibre optics.

## 3. Results

### 3.1. Immature Terrestrial Insects

#### 3.1.1. Introduction

The Purbeck terrestrial insect fauna is dominated in both abundance and diversity by Coleoptera (beetles), followed by Diptera (true flies) and Hemiptera (true bugs). Other orders, such as Orthoptera (‘grass’hoppers/crickets), Blattodea (cockroaches/cockroachoids) and Hymenoptera (wasps) are also regularly encountered. Some terrestrial taxa, mostly small, are preserved intact, having flown or been blown directly on to the water surface (e.g., some Diptera, Coleoptera, Thysanoptera (thrips) and Hymenoptera), or were probably shoreline scavengers with reduced transport involved (e.g., some cockroaches and beetles). Nevertheless, the majority of identifiable remains are detached adult wings and elytra probably transported from more-elevated gymnosperm-forested hinterlands.

The Wealden terrestrial entomofauna is also dominated by Coleoptera, but followed by Blattodea and Hemiptera, with Diptera less abundant like other insect orders and Thysanoptera unrepresented, suggesting somewhat more sorting of transported insect assemblages. Intact small insects in general are much less common than in the Purbeck, reflecting the somewhat more energetic fluvial-influenced depositional setting.

Being flightless and mostly fragile, the immature stages of terrestrial insects would have low chances of fossilisation in both the Purbeck and the Wealden; hence their body fossils are scarce and restricted to nymphal Hemiptera.

#### 3.1.2. Hemipteran Body Fossils

Most conspicuous among the immature Hemiptera (Homoptera) in both the Purbeck and Wealden are small oval, generally convex, segmented carapaces. These are evidently sessile nymphs of terrestrial taxa, since some are fusainised (see below) and occur in hypersaline deposits yielding no traces of aquatic insects.

Such comparatively small fossils are difficult to classify precisely. Superficially, however, they closely resemble immature instars of extant Aleyrodidae (whiteflies) in the hemipteran suborder Sternorrhyncha, which are mostly tropical sessile ‘parasites’ of vegetation, especially angiosperms but also ferns and even conifers [13], with mobile winged adults. Whitefly eggs are laid on the underside of leaves and hatch into a mobile ‘crawler’ instar that seeks out a suitable feeding site before transforming into the sedentary ‘scale’ stage (usually three instars) in which they continue feeding with their sap-sucking piercing mouthparts and eventually pupate [14]. Today, they can be serious crop pests, especially in greenhouses.

The Purbeck and Wealden fossils clearly display what appears to be a small rounded vasiform orifice, situated on the dorsal surface near the rear of the body, which among recent Hemiptera is unique to aleyrodids [14]. The vasiform orifice comprises the anus, a ‘lingula’ which expels excreta, and a covering ‘operculum’. Although the fossils do not preserve sufficient detail to elucidate all these sub-structures, and a dorsal opening alone is not unique to these insects, the gross morphology supports an aleyrodid-like interpretation of these fossils.

The extinct aleyrodid subfamily Bernaeinae was established for the earliest members of the family, known from adult material mostly from the Late Jurassic and Cretaceous of Asia [15]. Among these, however, is a Purbeck wing assigned to *Juleyrodes* sp. from a Durlston Formation horizon in Durlston Bay (in bed DB175; Figure 2) that has also yielded remains of these putative aleyrodid nymphs. Three such nymphs, designated Homoptera larvae A–C, were figured from the Purbeck and Wealden groups by Jarzembowski and Coram [16] and cited by Shcherbakov [15], although only non-aleyrodid sternorrhynchan adults have been described so far from the latter group [4].

**Figure 4:**
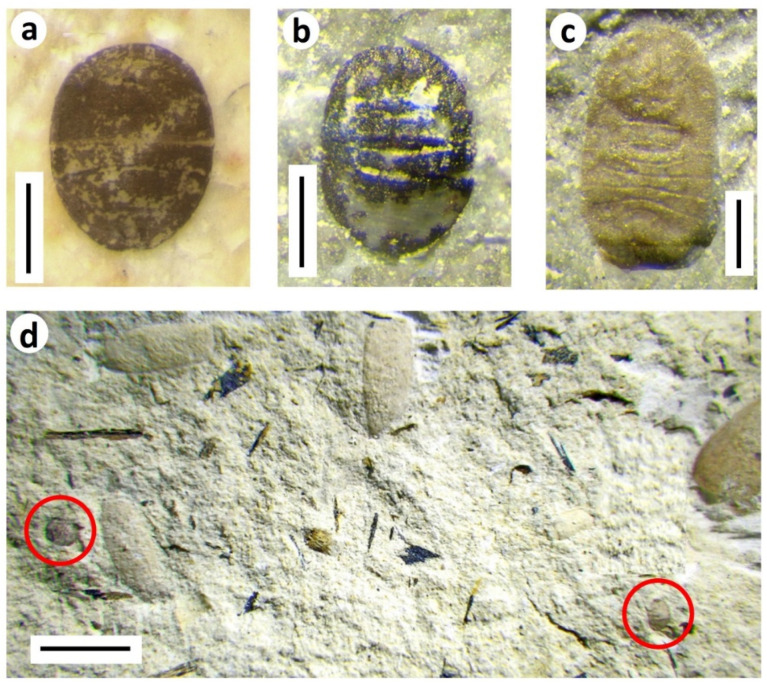
Aleyrodid–like terrestrial hemipteran nymphs from the Purbeck: (**a**) *Homopteron* A (BRSUG 29958.1), wetted with ethanol; (**b**) *Homopteron* A (BRSUG 29958.2), fusainised example; (**c**) *Homopteron* B (BRSUG 29958.3; ex. Mr. A.A. Mitchell). (**a**–**c**) from Durlston Formation of Durlston Bay. Scale bars 0.5 mm; (**d**) Examples of *Homopteron* A (circled) associated with charcoal and beetle elytra (BRSUG 29958.4), Lulworth Formation, Durlston Bay. Scale bar 5 mm.

The former line drawings of the two Purbeck morphs are supplemented here by photographs of different individuals (Figure 4). The commonest morph (A of Jarzembowski and Coram [16]) is sub-circular in shape (Figure 4a,b,d). It has a segmented abdomen and a distinctive lateral ‘band’ across the middle part of the body. Its size ranges from approximately 0.5–1.0 mm in length. The smaller carapaces tend to be distinctively convex and the segmentation less distinct so that they can be confused with tiny beetle elytra. These may represent a separate species from the larger individuals with more distinct segmentation, or may simply be an earlier instar. Morph A is relatively common and is encountered in most insect-bearing Purbeck horizons. It can be particularly abundant on charcoal-rich surfaces (Figure 4d), sometimes accompanied by the remains of wood-associated insects such as ‘cupedid’ beetles. It can therefore be surmised that these immature bugs lived on vegetation, either gymnospermous trees or understorey ferns, that were prone to fires, leading to the carapaces being blown or washed, along with burnt vegetation, into the depositional water bodies. This is supported by the fact that some of the carapaces, glossy black in appearance, appear to have been charcoalified (fusainised) (Figure 4b). In contrast to adpressions, rather than partially destroying the specimens, the process of fusainisation can serve to enhance preservation, enabling the finely pitted surface of the carapace, ordinarily not preserved, to be revealed through Scanning Electron Microscopy (figured in Coram and Jepson [17]).

The other morph (B) is much less common, known from just a few specimens (Figure 4c). It is somewhat larger (*c*. 1.5 mm) and more elongate, probably not as rigidly sclerotised, and with a distinctively crenelated margin. It is possible that it corresponds to a more advanced instar of morph A, although in recent whiteflies, sclerotization tends to increase with nymphal development [14], the reverse of what is seen here.

**Figure 5:**
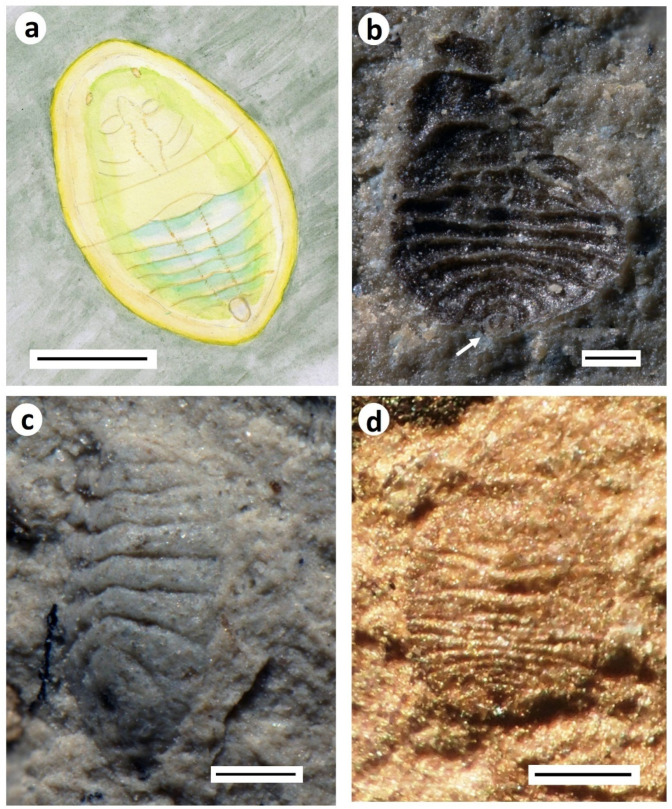
Aleyrodid-like terrestrial hemipteran nymphs from the Wealden Group: (**a**) Artist’s impression of *Homopteron* C, Weald Clay Formation, Auclaye brickworks, Surrey, UK (original, B. Jarzembowski); (**b**) *Homopteron* D, Weald Clay Formation, Smokejacks brickworks, Surrey, UK (NHMUK Pl II.3113). Arrow indicates lingula-like structure on dorsal orifice; (**c**) *Homopteron* E, Weald Clay Formation, Smokejacks brickworks, Surrey, UK (NHMUK Pl II.3112); (**d**) *Homopteron* F, Wadhurst Clay Formation, Ashdown brickworks (Bexhill), E. Sussex, UK, (BEXHM 2021.208.116; ex. Mr. A.A. Mitchell). Scale bars 0.2 mm.

Morph C of Jarzembowski and Coram [16], from the upper Weald Clay, is fairly poorly preserved, but resembles morph A from the Purbeck, differing in proportions and simplicity of the posterior end (Figure 5a).

The terms nymph (versus larva) and pupa (versus puparium for last instar) are still in widespread use and Homoptera are not necessarily regarded as a monophyletic group (e.g., [18]). We therefore refer the nymphs to the collective *Homopteron* Handlirsch, 1906 [19] and figure two additional forms found in sideritic concretions of the upper Weald Clay Formation at the Smokejacks brickworks, Surrey. *Homopteron* morph D (Figure 5b) notably differs from C, also from the upper Weald Clay, in the more advanced development of the wing pads; moreover, the dorsal orifice has a central longitudinal fold suggestive of a lingula. *Homopteron* morph E (Figure 5c), only known from the hind-body, has a more compact body form with accentuated sinuosity of the transverse segmental margins. A further form, *Homopteron* morph F, in a sideritic concretion from the Wadhurst Clay of the Ashdown brickworks, resembles A except that the head region appears shorter (Figure 5d). 

The UK nymphs resemble the bernaeid aleyrodoid (?) figured by Whalley and Jarzembowski [20] from the Early Cretaceous lithographic limestone of Montsech, Spain (Barremian like the upper Weald Clay). Displaying a dorsal operculum with associated ornamentation, and a marginal rim, this is now referred to as *Homopteron* morph G.

The sternorrhynchan identity of the Purbeck and Wealden fossils, we believe, is reasonably secure, although membership in another hemipteran group, perhaps now extinct, cannot be completely excluded. Their exact affinities await the discovery of better preserved material, such as inclusions in Wealden amber.

**Figure 6:**
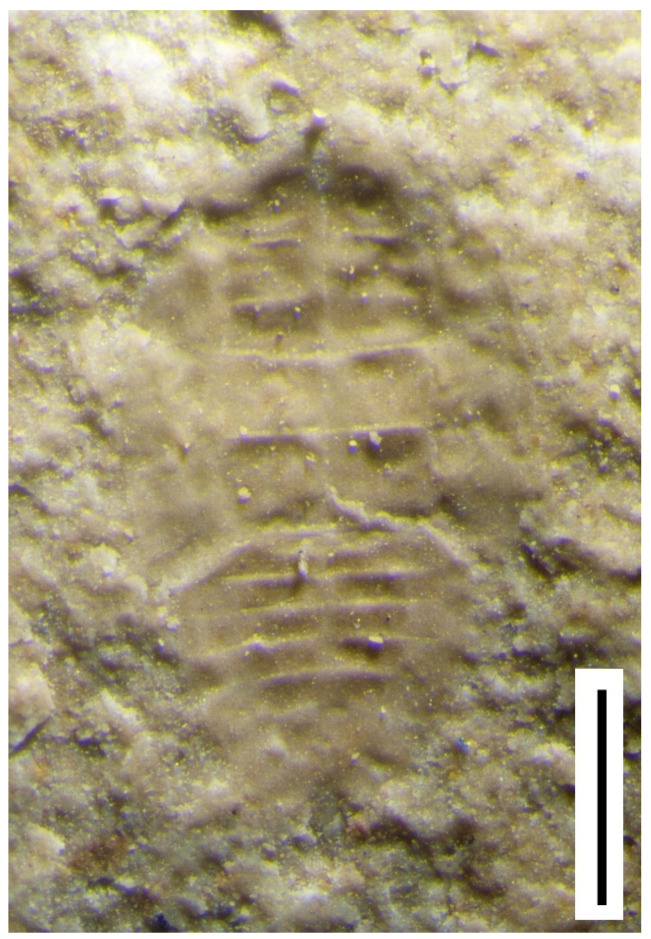
Possible nymphal terrestrial hemipteran (BRSUG 29958.5). Durlston Formation, Durlston Bay. Scale bar 1 mm.

Rare wingless bodies, such as that in Figure 6, may be transported nymphs of non-sessile terrestrial Hemiptera like progonocimicids [21]; however, in the absence of venation and other defining characteristics, a firm identity cannot be established.

#### 3.1.3. Trace Fossils

**Figure 7:**
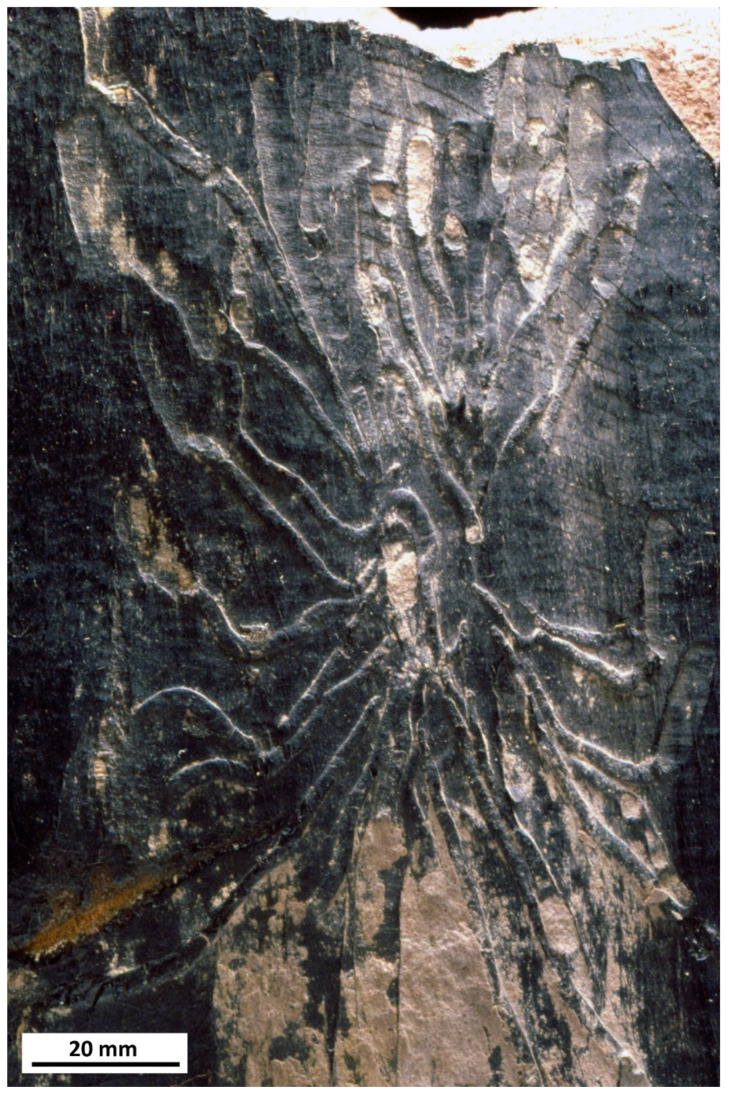
Larval beetle borings in wood: *Paleoscolytus sussexiensis* Jarzembowski, Wadhurst Clay Formation, Crowborough, E. Sussex, UK, NHMUK Pl In 41756 (modified after Jarzembowski [26]).

Uncommon trace fossils in the form of plant borings record the activities of immature terrestrial insects. The ichnospecies *Paleoscolytus sussexiensis* Jarzembowski, 1990 [22] from the lower Wealden is attributed to scolytine bark beetles [23]. These distinctive larval borings, in a more or less radial pattern from a central elongate egg chamber, were formed in coniferous wood (Figure 7); less conspicuous, associated borings suggest the presence of a second beetle, as is often the case in weakened trees susceptible to insect attack. 

Rare basal Purbeck examples of presumed larval beetle borings in coniferous wood are also known from Dorset and Wiltshire [17,24]

Finally, the possibility of insect borings associated with vertebrate remains is a work in progress [25].

### 3.2. Immature Aquatic Insects—Purbeck Group

#### 3.2.1. Introduction

Insects with aquatic development are important components of non-marine ecosystems today. Some spend much or most of their adult lives in the water (e.g., various bugs and beetles); others spend their entire emergent lives out of the water, although often remain close to it (e.g., dragonflies). 

Aquatic insect diversity declines with increasing salinity, with insect orders differing in their sensitivity; this has implications for the taxa preserved in the Purbeck and Wealden sediments. Thus, Plecoptera (stoneflies) and Ephemeroptera (mayflies) are restricted to fresh or near-fresh water [27]. Some Odonata (dragonflies), Trichoptera (caddisflies), aquatic Hemiptera (true bugs) and aquatic Coleoptera (beetles) can tolerate a range of brackish salinities up to normal marine, corresponding to a salt content of around 35 parts per thousand (e.g., larvae of caddisflies in the family Chathamiidae, which inhabit tidal pools in Australasia [28]), but only rarely higher salinities. Some flies (Diptera), in contrast, can thrive in hypersaline water [27]. In all orders, however, diversity falls steeply with increasing salinity, although abundance of individual species can be very high. Other factors clearly also affect aquatic insect occurrence (e.g., water physics and chemistry, vertebrate predation) but from a geological perspective, the key variable in low-lying deposits such as the Purbeck and Wealden was the proximity (or otherwise) of the sea [29].

#### 3.2.2. Taxa Present

Remains of insects with aquatic development can be abundant at certain levels in the Purbeck. In all, in excess of 100 taxa have been recognised [30], including, for example, over 20 described species of dragonflies [17]. The vast majority of these, however, are known only from disarticulated portions (mostly wings and elytra) of adult insects, with no immature representation. 

The Purbeck rocks were deposited in water bodies ranging from near-fresh through near-marine to highly hypersaline, depending on climate and the relative inputs of marine and fresh water [31]. As today, the majority of Purbeck insects with aquatic immatures would almost certainly have developed in fresh or near-fresh water. Such deposits, mostly seen in the higher part of the succession, are lithologically unsuitable for insect preservation since they are heavily bioturbated and often packed with coarse shelly debris. Purbeck insect fossils are therefore restricted to fine-grained fissile micrites deposited in brackish-hypersaline lagoonal settings, mostly seen in the Lulworth Formation (Figure 2). Preserved remains of adult insects with presumed freshwater aquatic larvae, like most terrestrial remains, are evidently transported and so generally similarly disarticulated.

Some adult aquatic insects, however, can be well-preserved, even though their immature stages are unknown. These include various beetles, particularly extinct coptoclavids [32] and hemipterans including still-extant naucorids (creeping water bugs) and belostomatids (giant water bugs) [33]. Being capable of flight, these are interpreted as insects that flew into water bodies too saline for aquatic development and then either promptly died or managed only to survive as adults. They may, alternatively, have been transported, alive or dead, from fresher water bodies, or even spring or stream-fed margins of the same water bodies, since in modern lagoonal settings salinities can vary greatly over short distances (e.g., [34]). The presence of rare transported freshwater molluscs such as unionoid bivalves in hypersaline Purbeck deposits [31] supports this scenario. 

Being flightless and generally more fragile, the stratigraphic distribution of immature aquatic insects in the Purbeck is more restricted than that of adult remains (see Figure 2 for Durlston Bay; all taxa other than Ephemeroptera are also found in the lowermost Lulworth Formation of the Isle of Portland). Taxa that have been recorded comprise the following.

**Figure 8:**
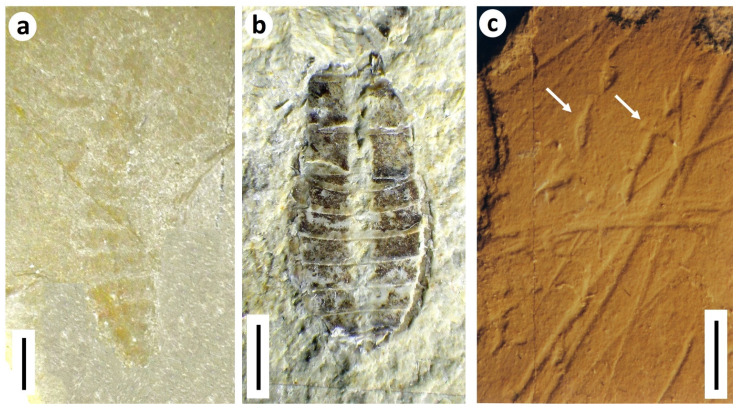
Purbeck odonatan (dragonfly) nymphs. (**a**) Ghost-like but essentially entire nymph from Lower Lulworth Formation, Durlston Bay (BRSUG 29958.6); (**b**) Better preserved but less complete example from the Durlston Formation, Durlston Bay (BRSUG 29958.7); (**c**) Parallel tracks (arrowed) perhaps left by an odonatan nymph, associated with linear grooves possibly produced by drifting plant material (BRSUG 29958.8). Scale bars 2 mm.

**Figure 9:**
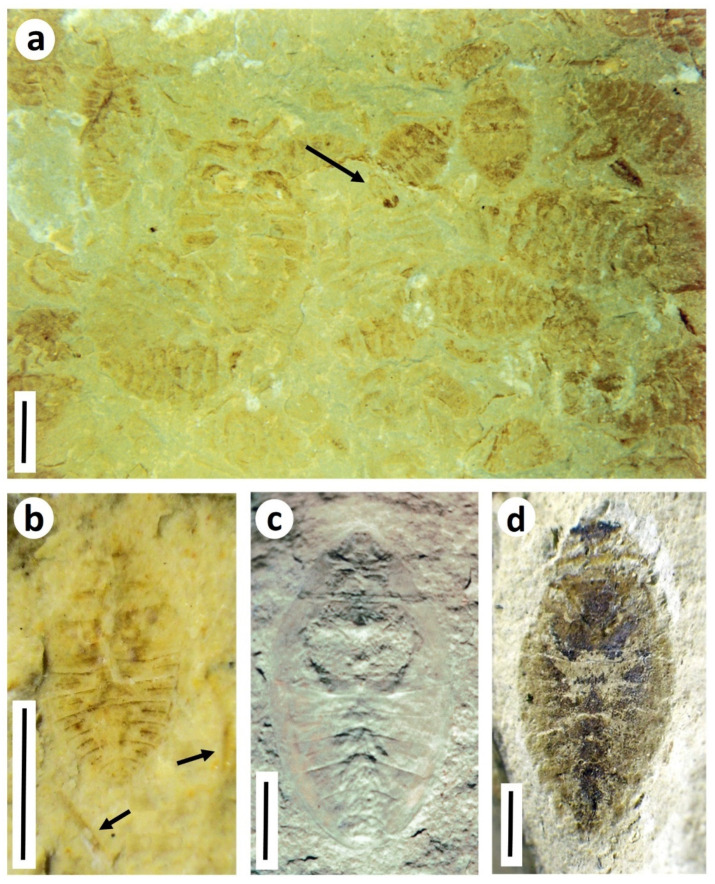
Purbeck Group aquatic Hemiptera (true bugs). (**a**) Massed nymphs of abundant aquatic bug *Nepidium stolones* Westwood, basal Purbeck, Durlston Bay, Dorset. There is also a solitary fly pupa (arrowed) (BRSUG 29958.9); (**b**–**d**) Life stages of *Nepidium*; (**b**) Early nymphal instar, arrows indicating swimming legs, Durlston Formation, Durlston Bay (BRSUG 29958.10); (**c**) Advanced nymphal instar. This is the holotype of the species, collected in the 1850s from the lower Lulworth Formation of Ridgeway, near Weymouth, Dorset and is now in the Sedgwick Museum, Cambridge (Fisher collection no. 37); (**d**) Adult, Lulworth Formation, Isle of Portland (BRSUG 29958.11). Scale bars 2.0 mm. (**a**,**c**) were previously figured in [17].

**Figure 10:**
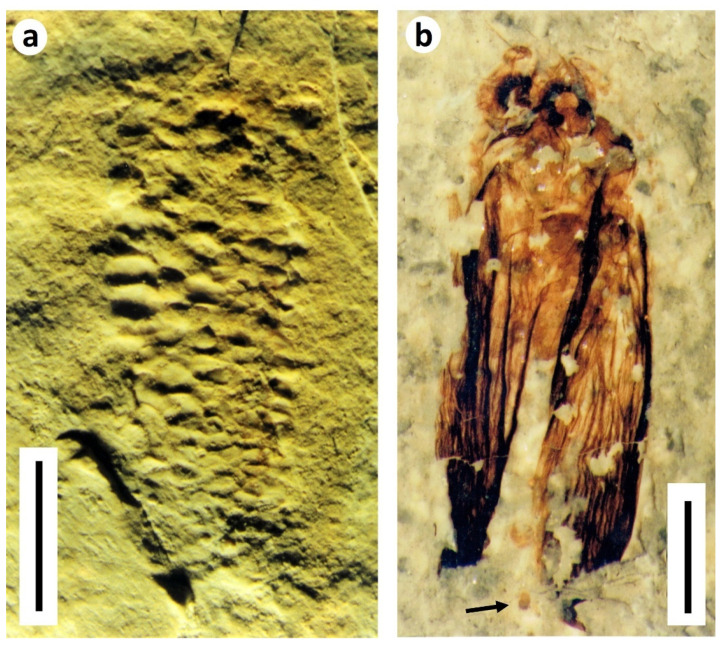
Purbeck Group Trichoptera (caddisflies). (**a**) Larval case made from ostracod valves (*Ostracindusia*), lower Lulworth Formation, Portland, Dorset (MNEMG 1999.36; previously figured in [3,17]); (**b**) Adult caddisfly presumed to have died in process of emergence. Probable remnants of pupal case arrowed. Durlston Formation, Durlston Bay, Dorset (MNEMG 22003.6; ex. Mr. A.A. Mitchell; previously figured in [17,40]). Scale bars 2 mm.

**Figure 11:**
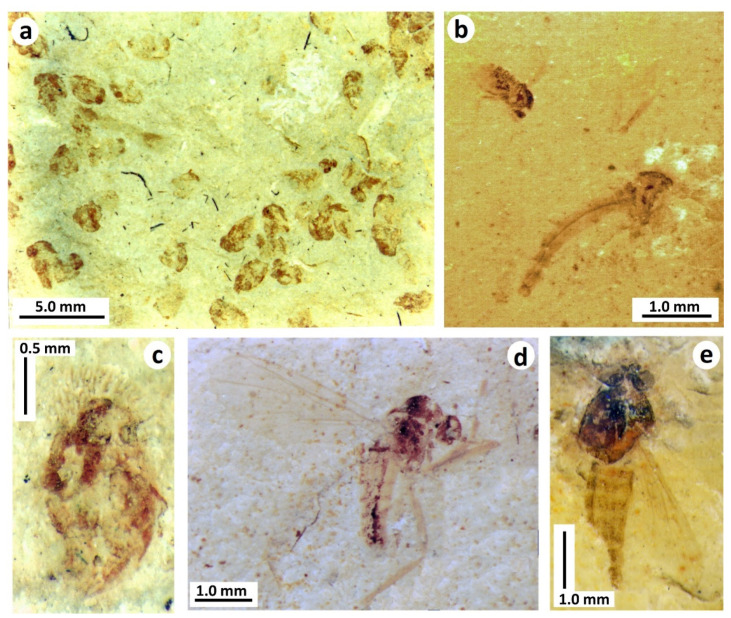
Purbeck Group Diptera (true flies). (**a**,**b**) Details of clusters of dipteran pupae and/or emergent adults, (**a**) from the Lulworth Formation of Portland (MNEMG 1999.34; previously figured in [17]); (**b**) from the Durlston Formation of Durlston Bay (MNEMG 1998.23; previously figured in [47]); (**c**) Simuliid-like pupa from slab in (**a**) showing respiratory filaments on top of head (previously figured in [3,17]); (**d**,**e**) Well-preserved adult flies from horizons at Durlston Bay containing dipteran pupae; (**d**) Chaoborid from Lulworth Formation (MNEMG 1998.25; previously figured in [17,47]); (**e**) Chironomid from Durlston Formation (BRSUG 29958.12).

- Ephemeroptera (mayflies). No nymphal remains of Ephemeroptera have been reported; however, a subimaginal wing (*Durlophlebia radleyi* Sinitshenkova and Coram, 2002 [35]) from the Lulworth Formation of Durlston Bay represents the sole record of this order in the Purbeck. A mayfly subimago, popularly known as a ‘dun’, is a winged intermediate stage between nymph and adult that is capable of flight.
- Odonata (damselflies and dragonflies). Fossils of Odonata, mostly adult wings, are conspicuous and diverse elements of the Purbeck entomofauna, and include representatives of the two extant suborders Zygoptera and Epiprocta (including Anisoptera, or ‘true’ dragonflies), along with rare examples of extinct Archizygoptera and Tarsophlebioptera [17]. Fossils of nymphs (or their ecdysed cuticles) are much less common, and are usually indistinctly preserved, although often fairly intact [16,17] (Figure 8a,b). Among extant Odonata, the Purbeck fossils most resemble those of Anisoptera (which dominate the preserved adult fauna), being relatively wide-bodied and lacking evidence of the prominent caudal lamellae seen in Zygoptera. There are at least two morphotypes of nymph present, although it cannot be determined at present which taxa known from adult material they might belong to. Parallel discontinuous tracks observed rarely on bedding surfaces in a Durlston Formation horizon (DB175; Figure 8c) have been tentatively attributed, on the basis of their size and the presence of immature remains in the same bed, to odonatan naiads [30].
- Hemiptera (true bugs). Aquatic hemipteran nymphs can be commonly preserved in certain Purbeck horizons, occasionally massed on bedding planes (Figure 9a), reflecting mass mortality events or hydrodynamic concentration of ecdysed cuticles. All well-preserved specimens can be referred to *Nepidium stolones* Westwood, 1854 [36], winged adults of which commonly occur alongside the nymphs. Here we supplement the line drawings of selected life stages in Coram and Jarzembowski; Figure 6 [3] with photographs of specimens (Figure 9b–d). *Nepidium* Westwood, 1854 is of uncertain heteropteran family affinities, although well-preserved individuals show a distinctive pair of elongate ‘rowing legs’ in common with recent Notonectidae (backswimmers) and Corixidae (water boatmen).
- Trichoptera (caddisflies). Larval cases, mostly composed of ostracod valves (igen. *Ostracindusia* Vialov, 1973 [37]) occur low in the Lulworth Formation of Durlston Bay and the Isle of Portland [3] (Figure 10a). Indistinct cases are also known from the Durlston Formation, but these appear to be composed of faecal pellets (*Coprindusia* Ivanov, 2006 [38]). Occasional fossils of exquisitely-preserved but crumpled-winged adult caddisflies from both Lulworth and Durlston formations are interpreted as newly pupated individuals which failed to emerge at the water surface [17,39,40]. Some, in fact, show what appear to be remnants of the pupal cases at the rear of the abdomen (Figure 10b). Eleven species of Trichoptera have been described from the Purbeck on the basis of adult wings, the fossils being numerically dominated by members of the stem genus *Purbimodus* Sukatsheva and Jarzembowski, 2001 [41], which survived into the Wealden. The presumed teneral caddisflies, despite their often fine preservation, cannot be linked to known taxa because the crumpled wings obscure important venational details. In other respects, however, they are morphologically indistinguishable. Intraspecific variation in choice of case building material in response to local availability has been documented in recent caddisflies [42], raising the possibility, as suggested by Coram and Jarzembowski [3], that a single opportunistic Purbeck species may have produced cases assignable to two different ichnogenera, making use of whatever case-building materials were available (ostracods being largely absent from the horizon with faecal-pellet cases).
- Diptera (true flies). Small aquatic pupae of nematoceran Diptera can be abundant at certain levels (Figure 11a–c). They can also be accompanied by adult flies which usually have indistinct crumpled wings, suggesting that these were newly pupated individuals that failed to emerge from the water, as suggested for the caddisflies above. Their taxonomic identity is uncertain, however, at least some and perhaps all are likely to be chironomids or chaoborids since both families are also known from well-preserved adult specimens that presumably emerged locally (Figure 11d,e). A chironomid identity is probably more likely since recent chaoborids, in contrast to chironomids, are intolerant of elevated salinities [43] and thus are less likely to have thrived in the saline Purbeck waters the pupae are preserved in (see discussion below). The total number of preserved immature dipteran species cannot be determined at present, but overall diversity may have been low since there is limited morphological diversity evident. Nevertheless, other nematoceran families known from adult material could be present, including small Limoniidae (craneflies, common in the Purbeck) and Ceratopogonidae (biting midges, known from a single Purbeck wing [44]). The pupa in Figure 11c uniquely bears hair-like respiratory filaments on its head, which, along with its general body proportions most closely resemble Simuliidae (black flies) among recent Diptera with aquatic development. Simuliidae, which can tolerate brackish salinities [45], have a fossil record extending back to the Jurassic [46] although no adult wings have to date been recognised in the Purbeck.

### 3.3. Immature Aquatic Insects—Wealden Group

#### 3.3.1. Taxa Present

**Figure 12:**
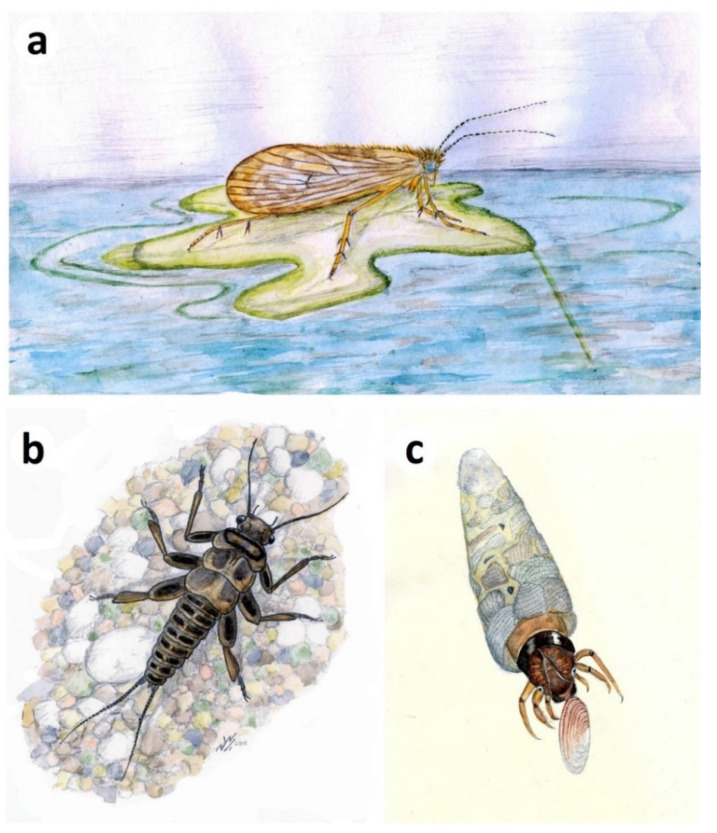
Artists’ impressions of Wealden aquatic insects. (**a**) Adult *Purbimodus minor* Sukatsheva and Jarzembowski, 2001 [39], resting on *Bevhalstia pebja* Hill, 1996 [48], Weald Clay (although the caddisfly, unlike the plant, also occurs in the Durlston Formation of the Purbeck Group), forewing length *c*. 9 mm (original, B. Jarzembowski); (**b**) Plecopteran nymph *Ecdyoperla fairlightensis* Sinitshenkova, 1998 [12], Ashdown Formation, Fairlight, E. Sussex, UK, body length 9 mm (modified after Jarzembowski et al. [49]); (**c**) *Conchindusia rasnitsyni* Jarzembowski, 1995 [50], Ashdown Formation, Hastings, E. Sussex, UK, case length (average) 17 mm (original, B. Jarzembowski).

Adult aquatic insects, usually fragmentary, are fairly common at some levels, especially in the upper Wealden (Weald Clay Formation), comprising Odonata and less frequent Trichoptera (Figure 12a), Hemiptera (including belostomatids, naucorids), Coleoptera (including schizophorids, hydrophilids), and Diptera (including chironomids and chaoborids [4]). 

Aquatic immature insects are more restricted and found mainly in the lower Wealden. Orders present are as follows.

- Plecoptera (stoneflies). The lower Wealden has produced a unique plecopteran nymph (Figure 12b). *Ecdyoperla fairlightensis* Sinitshenkova, 1998 [12] was recovered from the Fairlight clay facies in the lower Ashdown Formation of Fairlight, near Hastings (Figure 1 and Figure 3). It is the only European Cretaceous representative of this order recorded to date and the family placement is problematic.
- Ephemeroptera (mayflies). The upper Weald Clay Formation of Smokejacks brickworks, Surrey, has produced a unique mayfly nymph, *Schistonotorum wallisi* Jarzembowski and Wang, 2019 [51]. Adult wings are unknown. Sedimentary burrows of the ichnogenus *Rhizocorallium* Zenker, 1836 [52] from the lower Wealden Wadhurst Clay Formation have been attributed to burrowing mayfly nymphs [53].
- Trichoptera (caddisflies). The lower Wealden river-channel deposits of the upper Ashdown (Sand) Formation at Hastings [54] have produced a diverse trichopteran indusifauna characterised by the extinct ichnogenus *Conchindusia* Vialov and Sukatsheva, 1976 [55], utilising ‘conchostracan’ (clam shrimp/spinicaudatan) carapaces (Figure 12c), and for which there appears to be no exact modern analogue. A comparatively minor channel deposit, the Northiam Sandstone, in the overlying Wadhurst Clay Formation has recently yielded an indusifauna reported below. Caddisfly cases also occur sporadically in the upper Wealden, small numbers being found in the Weald Clay Formation; amongst these may be mentioned the ichnogenus *Piscindusia* Jarzembowski, 1995 [50], also reported from scour fills in the Wadhurst Clay Formation and the Vectis Formation (coeval with the youngest Weald Clay) of the Isle of Wight [4], as there appears to be no modern analogue incorporating fish scales/bones in case construction, any more than clam shrimps [4].

#### 3.3.2. Northiam Sandstone Indusifauna

**Figure 13:**
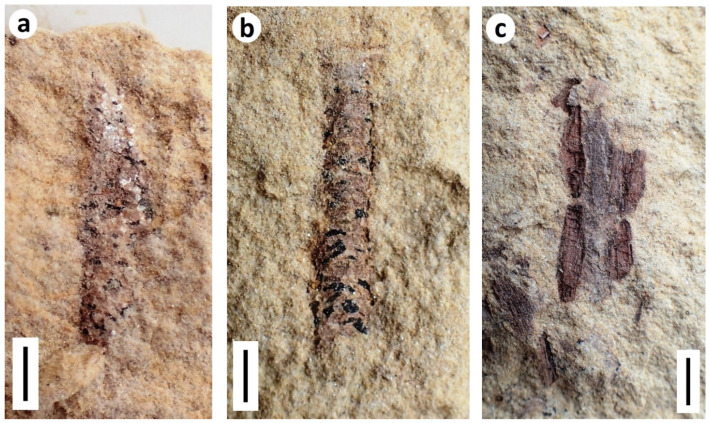
Caddisfly larval cases from Northiam Sandstone Member, Bexhill, E. Sussex, UK. (**a**) *Folindusia* Berry, 1927 [59] (BEXHM 2021.208.19), showing mineral grains (shiny) in initial portions; (**b**) *Folindusia* sensu stricto (BEXHM 2021.208.105); (**c**) *Folindusia* with well-developed edging (BEXHM 2021.208.31). Scale bars 2 mm.

The new indusifauna, on 115 soft pale siltstone/fine sandstone blocks, some with multiple specimens, was collected by Peter and Joyce Austen (2001–2017) and is from the mid-Valanginian Northiam Sandstone Member in the upper Wadhurst Clay Formation of the Ashdown brickworks, near Bexhill, East Sussex, UK (NGR TQ 720095). The member is currently interpreted as representing minor (3m thick) river-channel deposits [56]. It is therefore sedimentologically similar to the upper Ashdown Formation, which has yielded an older indusifauna at East Cliff, Hastings, E. Sussex [54]. The general flow direction in both outcrops was northeasterly off the Londinian Massif [57]. It was also sluggish, here mildly alkaline rather than acid, but essentially fresh (not exceeding oligohaline), shallow water with few fish, and cryptogams sometimes on bordering bars [58]. Unlike at East Cliff, however, the indusifauna is composed almost exclusively of *Folindusia* ichnospecies, accompanied by a single *Terrindusia* Vialov, 1973 [37] specimen, morphotaxonomically distinct but actually the younger stage of a *Folindusia* Berry, 1927 [59] (Figure 13a). Both simple and edged cases are represented, suggesting that at least two subichnogenera are present (Figure 13b,c). The indusifauna therefore resembles that of Late Jurassic/Early Cretaceous Chernovskie Kopi in Siberia rather than Hastings in East Sussex; the Transbaikalian assemblage, however, still needs to be studied in detail [60]. It may be added that a few younger caddisfly cases have been reported ‘upstream’ nearer Londinia in the upper Weald Clay of Kent, and these too belong to *Folindusia* [61].

## 4. Discussion

Purbeck and Wealden immature terrestrial insect body fossils are restricted to transported hemipteran remains which contribute little to our knowledge of terrestrial palaeoenvironments and ecosystems. In contrast, the immature aquatic insects, although relatively undiverse, are represented by several orders and in many cases have undergone minimal transport. They thus have the potential to provide important information about the aquatic settings in which they lived.

The total diversity of Purbeck autochthonous immature aquatic insects is uncertain, but is evidently much less than that of adult insects with aquatic immature stages, which also include, in addition to the four orders listed earlier, Coleoptera (beetles), including Coptoclavidae, Dytiscidae and Gyrinidae [32], which are not represented by recognisable preserved larvae.

This disparity in preserved diversity is likely linked in part to the greater fragility of immature insects, their often-indistinct preservation and the fact that, lacking functional wings, they are more difficult to distinguish as separate species. This reflects the general challenge of studying immature insect fossils; historically, studies have focused on imaginal remains, especially wings. 

Undoubtedly most important, however, was the salinity of the water bodies that preserved the insect remains. The single Purbeck mayfly ‘dun’ was found in a halite-bearing deposit, indicating at least occasional excursions into hypersalinity and water conditions unsuitable for larval development; thus, this insect was most likely transported (or flew) from a more benign habitat elsewhere. Ephemeroptera and Plecoptera (stoneflies), in addition to being virtually restricted to fresh water, have poor dispersal ability (e.g., compared to dragonflies), potentially explaining their scarcity and absence, respectively, in the Purbeck fossil record.

The remaining immature aquatic taxa can often be preserved intact, suggesting they were buried more-or-less where they lived and died, i.e., are autochthonous. This is supported in the case of the *Nepidium* nymphs and dipteran pupae because these can also be found clustered in large numbers in some horizons. Elevated water salinity is supported by the nature of the biota preserved alongside them, which lacks obligatorily fresh or normal marine organisms, but includes taxa such as the gastropod *Hydrobia* Hartmann, 1821 [62], recent species of which tolerate a wide range of brackish salinities up to normal marine (e.g., [63]).

The immature aquatic taxa actually present in any particular bed can vary, however, probably in response to factors including standing salinity. The most diverse aquatic immature assemblage contains all the autochthonous taxa listed earlier (*Nepidium* + Diptera + Odonata + Trichoptera). *Nepidium* nymphs and/or dipteran pupae dominate, their relative proportions often varying between different laminae within the same bed, indicating either subtle differences in local water conditions or the ‘capture’ of different stages of insect breeding cycles. Odonata and Trichoptera, although present, are always comparatively uncommon. This assemblage is found low in the Lulworth Formation of Durlston Bay and the Isle of Portland and in the Durlston Formation of Durlston Bay (Figure 2). Mesohaline salinities (mid brackish, 5–18 parts per thousand based on the ‘Venice System’ [64]) are suggested from comparison with recent settings in which Plecoptera and Ephemeroptera are absent and insect species diversity is similar to that suggested by the Purbeck fossil evidence (e.g., 10 species recorded in UK saltmarsh pools with salinities up to 10 parts per thousand [65]).

This most diverse Purbeck immature aquatic insect assemblage can also be accompanied by small leptolepid fish, often well-preserved and presumably autochthonous. These fish are likely to have preyed on immature aquatic insects, since presumed fish coprolites from Portland contain remains of dipteran pupae within them [30]. Larger fish, turtles and crocodilians may have been present too, although their remains in beds yielding immature aquatic insects are rare and fragmentary, and so may have been transported from other habitats.

Terrestrial plant material other than charcoal is scarce in those beds yielding this ‘diverse’ assemblage, suggesting that it did not form a large part of the insects’ diets. The rock can, however, be stromatolitic (microbialitic) in appearance [47], suggesting the presence of algal biomats, which may have been the preferred food of immature Diptera and Trichoptera (as well as perhaps trapping some individuals before they could emerge as adults from the water surface). The diet of the *Nepidium* bugs is uncertain, although preserved gut contents are structureless under SEM examination, suggesting that the food was either liquid or extremely fine particulate [30]. In keeping with most recent aquatic Heteroptera, they may well have been piercing/sucking predators of other aquatic organisms, including fly larvae, although, if of corixid affinities, they could instead have consumed algae and organic detritus [66]. The odonatan nymphs, as they are today, would have been predatory, snatching aquatic insects and other prey with a hinged modified mouthpart (labium or ‘mask’).

A less diverse immature aquatic insect assemblage, seen at several levels in the Lulworth Formation of Durlston Bay (Figure 2), contains *Nepidium* nymphs and dipteran pupae, but no recorded Odonata or Trichoptera. There are also horizons in the topmost Lulworth and Durlston formations of Durlston Bay in which *Nepidium*, too, is apparently absent, leaving only Diptera.

These restricted assemblages may reflect progressive increases in standing salinity along the spectrum of polyhaline (high brackish: 18-30 parts per thousand) to euhaline (normal marine: 30-40 parts per thousand). Dipteran pupae in bed DB178 in the Durlston Formation (Figure 2) are preserved alongside molluscs such as oysters and pectinid bivalves commensurate with such salinities [67]. Whether or not these pupae represent different species to those found in the more diverse presumed mesohaline assemblage or represent one or a few euryhaline taxa is not presently known. The flies may even have been tolerant of a degree of hypersalinity (>40 parts per thousand), since some recent chironomids have extremely broad salinity tolerances ranging up to over 100 parts per thousand [68].

Unlike sediments containing the most diverse immature aquatic insect assemblages discussed above, the more restricted assemblages comprising *Nepidium* + Diptera or Diptera alone often contain the evaporites gypsum and halite. These precipitate at salt concentrations of 124 and 370 parts per thousand, respectively; the latter, in particular, almost certainly a salinity beyond what could be tolerated by aquatic insects. These evaporites, therefore, almost certainly precipitated in conditions (perhaps seasonal) when no insects were present, or formed displacively after the beds had been deposited. What they do indicate, however, is that the original source of the water in which these insect fossils are found was marine, with salinities at times reduced by the input of meteoric water, and at others elevated to hypersalinity through evaporation. They also suggest that habitat instability, in addition to standing salinity, could have had a large effect on aquatic insect diversity. There is some evidence that at least some of the Purbeck water bodies with less diverse faunas were relatively small (the beds being thin and of limited lateral extent), where diversity-depressing fluctuations in salinity, temperature, water depth and oxygen levels are more likely to have come into play [30]. Germane in this respect is that the less diverse insect assemblages (i.e., lacking immature Odonata and Trichoptera) are often accompanied by well-preserved examples of the isopod *Archaeoniscus* Milne-Edwards, 1843 [69], presumed to have been a shoreline/shallow-water inhabitant (e.g., bed DB107 on Figure 2).

Taking confounding factors such as habitat instability into account, immature aquatic insects remain a useful tool for establishing palaeosalinities within the brackish spectrum, complementing others that have been used for the Purbeck such as evaporites, oxygen and carbon isotopes, and other organisms such as molluscs (e.g., [70]). Like the insects, none of these are totally reliable in isolation, but are valuable as part of an integrated approach.

Unlike in the Purbeck, deposits of similar age with diverse fresh or near-fresh immature aquatic insect assemblages in which larvae of mayflies, stoneflies or coptoclavid beetles are often conspicuous or dominant are known elsewhere in the world, particularly in Asia. Preservation in these cases often resulted from deeper water leading to anoxic bottom conditions, suppressing the bioturbation that destroyed insect fossils in the shallow Purbeck water bodies. The immature insects that are preserved either lived in the water column (e.g., fly pupae and swimming bugs) or were transported a relatively short distance from shallower, oxygenated waters. An example of such a deposit is the Upper Jurassic Shar Teg assemblage from Mongolia [71].

Restricted fossil assemblages more comparable to the Purbeck are also known. In Upper Jurassic lacustrine deposits of Karatau, Kazakhstan, the only autochthonous immature aquatic insects found alongside the dominant corixid bug are limoniid (crane-fly) pupae [72]. Along with abundant terrestrial insect remains, these deposits have likewise yielded a single, presumably transported, mayfly wing. Brackish salinities are similarly implicated for the depressed aquatic insect diversity [72].

Recent low-diversity aquatic insect faunas similarly dominated by one or a small number of water bug species can be found, for example, in Mexican saline lakes [73].

The preserved Wealden immature aquatic insect fauna differs from that of the Purbeck in including a plecopteran but lacking Odonata, Hemiptera and Diptera, pointing to flow sensitivity [74], although diverse adult remains of these occur [4]. With the exception of the caddisfly case (indusi-) fauna, which suggests a trichopteran diversity greater than that represented by adult remains, the immature diversity is thus generally much lower than that of known adults, as in the Purbeck Group.

The Wealden climate was somewhat moister than the Purbeck and marine influence more limited, hence near-marine or hypersaline water conditions were infrequently experienced [75] and lack insect fossils (e.g., the so-called quasi-marine (polyhaline) bands). Insect fossils are mainly in fresh-brackish ‘mudplain’ (lake/lagoon) sediments (especially in the Weald Clay Formation), although aquatic insects (caddisfly cases) can be locally common in the freshwater ‘sandplain’ (river) deposits (Ashdown Formation). Salinity was therefore less likely to be a control on preserved immature insect diversity than in the Purbeck. Probably more significant was the generally more energetic fluvial depositional regime, which would have led to the destruction of delicate larval remains as well as the deposition of widespread sandstone facies unsuitable for insect preservation other than durable, multipurpose caddisfly cases (used for protection, camouflage and respiration). The rarity of immature body fossils in the finer-grained lacustrine mudstones is nonetheless puzzling and may reflect other environmental factors such as eutrophication [76]. 

The rare stonefly and mayfly nymphs from the Wealden are casual records that are not supplemented by adult remains. The slow currents and warm waters of the Wealden mudplain environments are likely to have been unconducive to substantial populations of these orders [51], as is the somewhat insular nature of the Wealden archipelago, which would have hampered dispersion and colonisation [12].

As in the Purbeck, the EPT index (Ephemeroptera-Plecoptera-Trichoptera) is skewed to the right, with dominance of trichopteran taxa [54], pointing to wide tolerance of multiple environmental stresses [77] possibly including nutrient availability [78]. The Purbeck additionally has a high proportion of Chironomidae, indicative of stressful water conditions [79]. In general, this underlines the paralic setting of southern England in the Early Cretaceous (in an archipelagic rift-basin), in contrast to the limnic developments of eastern Eurasia, as seen in the Chinese Jehol biota (back-arc volcanic lakes [50]).

Concentrations of caddisfly cases from the Tithonian Morrison Formation of the western U.S.A. are inferred to have been deposited by river floods into floodplain ponds [80], a taphonomic scenario similar to that seen in the Wealden with its residual entomofauna, although associated with volcanic ash rather than seasonal wildfire as in the latter.

## 5. Conclusions

Each of the several hundred insect species recognised from adult material in the Purbeck and Wealden groups of southern England would have had immature stages. Very few of these, however, have been preserved and they have received limited systematic attention due to often-indistinct preservation and the absence of diagnostic features, particularly venational.

Regarding terrestrial insects, those known as adults are most likely only a subset of those that lived in the local environment [3]. Most of these remains are disarticulated, reflecting various taphonomic pressures, which would have been particularly severe for wingless and often-fragile immature stages. In the Purbeck, damage was likely to have resulted from transport across broad sabkhas or mudflats that separated vegetated terrain from depositional water bodies [40], and in the Wealden across sandplains as well as mudflats, but not sabkhas, accompanied by disarticulating decay. Terrestrial immature body fossils are restricted to hemipteran remains. The great majority of these are the durable cuticles of sessile whitefly-like sternorrhynchan nymphs, which reveal a greater diversity than suggested by the solitary record of a putative adult wing.

The remains of immature aquatic insects can be locally abundant since they tended to live in the water bodies in which they were preserved, so transport was probably often negligible. Their distribution was, however, restricted by lithological and environmental factors. Thus, in arenaceous Wealden sediments, immature remains are often confined to durable caddisfly cases [54]. In the Purbeck, immature aquatic insects are absent from freshwater deposits, which are bioturbated and often shell-packed, and also from hypersaline settings in which few, if any, local insects would have survived. Preserved diversity is thus much lower than that indicated by transported adult remains. Nevertheless, these fossils provide information on the faunal composition and palaeoecology of brackish-water settings that would be otherwise unavailable.

## Data Availability

All data contained within the article.
